# Equilibrium States in Two-Temperature Systems

**DOI:** 10.3390/e20030183

**Published:** 2018-03-09

**Authors:** Evaldo M. F. Curado, Fernando D. Nobre

**Affiliations:** Centro Brasileiro de Pesquisas Físicas and National Institute of Science and Technology for Complex Systems, Rua Xavier Sigaud 150, Urca, Rio de Janeiro 22290-180, Brazil

**Keywords:** nonlinear Fokker-Planck equations, generalized entropies, nonextensive thermostatistics

## Abstract

Systems characterized by more than one temperature usually appear in nonequilibrium statistical mechanics. In some cases, e.g., glasses, there is a temperature at which fast variables become thermalized, and another case associated with modes that evolve towards an equilibrium state in a very slow way. Recently, it was shown that a system of vortices interacting repulsively, considered as an appropriate model for type-II superconductors, presents an equilibrium state characterized by two temperatures. The main novelty concerns the fact that apart from the usual temperature *T*, related to fluctuations in particle velocities, an additional temperature θ was introduced, associated with fluctuations in particle positions. Since they present physically distinct characteristics, the system may reach an equilibrium state, characterized by finite and different values of these temperatures. In the application of type-II superconductors, it was shown that θ≫T, so that thermal effects could be neglected, leading to a consistent thermodynamic framework based solely on the temperature θ. In the present work, a more general situation, concerning a system characterized by two distinct temperatures $\theta_{1}$ and $\theta_{2}$, which may be of the same order of magnitude, is discussed. These temperatures appear as coefficients of different diffusion contributions of a nonlinear Fokker-Planck equation. An H-theorem is proven, relating such a Fokker-Planck equation to a sum of two entropic forms, each of them associated with a given diffusion term; as a consequence, the corresponding stationary state may be considered as an equilibrium state, characterized by two temperatures. One of the conditions for such a state to occur is that the different temperature parameters, $\theta_{1}$ and $\theta_{2}$, should be thermodynamically conjugated to distinct entropic forms, $S_{1}$ and $S_{2}$, respectively. A functional $\Lambda \left[P\right]\equiv \Lambda (S_{1}\left[P\right],S_{2}\left[P\right])$ is introduced, which presents properties characteristic of an entropic form; moreover, a thermodynamically conjugated temperature parameter $\gamma (\theta_{1},\theta_{2})$ can be consistently defined, so that an alternative physical description is proposed in terms of these pairs of variables. The physical consequences, and particularly, the fact that the equilibrium-state distribution, obtained from the Fokker-Planck equation, should coincide with the one from entropy extremization, are discussed.

## 1. Introduction

The linear Fokker-Planck equation (FPE) represents one of the most important equations of nonequilibrium statistical mechanics; it describes the time evolution of a probability density $P(\vec{x},t)$ for finding a given particle at a position $\vec{x}$, at time *t*, diffusing under an external potential [1,2,3,4]. In the absence of external potential, the FPE reduces to the linear diffusion equation, usually associated with the description of the Brownian motion; a confining external potential yields the possibility of a stationary-state solution for a sufficiently long time. A particular interest in the literature is given to a harmonic confining potential, which leads to a Gaussian distribution as the stationary-state solution of the FPE [3,4].

It is very frequent nowadays, particularly within the realm of complex systems, to find dynamical behavior that falls out of the ambit of linear diffusion, usually called anomalous diffusion [5]. As typical examples, one may mention diffusion in media characterized by randomness, porosity, heterogeneity, as well as systems characterized by cooperative interactions among internal components, self-organization, and long-time memory. For dealing with these phenomena, one commonly uses a nonlinear (power-like) diffusion equation, known in the literature as porous-media equation [6]. Similarly to the linear case, by adding a confining potential one obtains a nonlinear Fokker-Planck equation (NLFPE) [7], as introduced in [8,9]. For a harmonic confining potential, this NLFPE presents a *q*-Gaussian distribution, typical of nonextensive statistical mechanics [10,11], as its stationary-state solution. In this way, the NLFPE introduced in [8,9] is associated with Tsallis entropy $S_{q}$ [12] (where q∈ℜ is called entropic index), since the *q*-Gaussian solution coincides with the distribution that maximizes $S_{q}$.

Considering that statistical mechanics may be formulated by starting from a given statistical entropy [1,10], many entropic forms were introduced since the proposal of $S_{q}$, as attempts to generalize the standard Boltzmann-Gibbs (BG) formulation. Among those many, we may mention the entropic forms of [13,14,15,16,17,18,19,20,21,22,23,24]; a pedagogical and comprehensive classification of entropic forms is given in [21], whereas a discussion of how the volume of phase space defines its associated entropy may be found in [22]. Additionally, the connections of NLFPEs with nonadditive entropic forms were explored through generalized formulations of the H-theorem [7,25,26,27,28,29,30,31,32,33,34,35,36,37,38,39,40,41,42,43,44,45,46,47] and particularly, the NLFPE of [8,9] is also related to the entropy with $S_{q}$ by an H-theorem.

Although one may pursue an analysis in arbitrary dimensions, by considering a probability density $P(x_{1},x_{2},\cdots ,x_{N},t)$, like those of [37,48,49,50], herein for simplicity, we will restrict ourselves to a one-dimensional space, described in terms of a probability density P(x,t). In this case, a general NLFPE may be defined as [32,33,51]

(1) $$\frac{\partial P(x,t)}{\partial t}=-\frac{\partial }{\partial x}\left\{A\left(x\right)\Psi \left[P\left(x,t\right)\right]\right\}+D\frac{\partial }{\partial x}\left\{\Omega \left[P\left(x,t\right)\right]\frac{\partial P(x,t)}{\partial x}\right\}\;,$$

where *D* represents a diffusion coefficient with dimensions of energy divided by the viscosity coefficient, and the external force A(x), with dimensions of force divided by the viscosity, is associated with a confining potential ϕ(x) [A(x)=−dϕ(x)/dx]. Herein, from now on, we will consider for simplicity the viscosity coefficient equal to one. The functionals Ψ[P(x,t)] and Ω[P(x,t)] should satisfy certain mathematical requirements, e.g., positiveness and monotonicity with respect to P(x,t) [32,33]; moreover, to ensure normalizability of P(x,t) for all times one must impose the conditions,

(2) $${P\left(x,t\right)|}_{x\to \pm \infty }=0\;;\;{\left.\frac{\partial P(x,t)}{\partial x}\right|}_{x\to \pm \infty }{=0\;;\;A\left(x\right)\Psi \left[P\left(x,t\right)\right]|}_{x\to \pm \infty }=0\;\left(\forall t\right)\;.$$

The NLFPE of Equation (1) recovers some well-known cases, as particular limits: (i) The linear FPE [1,2,3,4] for Ψ[P(x,t)]=P(x,t) and Ω[P(x,t)]=1; (ii) The NLFPE introduced in [8,9], associated with nonextensive statistical mechanics, for Ψ[P(x,t)]=P(x,t) and $\Omega \left[P\left(x,t\right)\right]=\mu {\left[P\left(x,t\right)\right]}^{\mu -1}$, where μ represents a real number, related to the entropic index through μ=2−q. It should be mentioned that a large variety of NLFPEs, like the one related to nonextensive statistical mechanics, or in the general form of Equation (1), or even presenting nonhomogeneous diffusion coefficients in the nonlinear diffusion term, have been derived in the literature by generalizing standard procedures applied for the linear FPE [1,2,3,4], e.g., from approximations in the master equation [37,46,51,52,53,54,55], from a Langevin approach by considering a multiplicative noise [46,56,57,58,59,60,61,62,63], or form methods using ensembles and a projection approach [64].

Almost two decades ago, NLFPEs presenting more than one diffusive term appeared in the literature [51,52,65,66], and particularly, very general forms were derived by considering the continuum limit in a master equation with nonlinear transition probabilities [51,52]. A special interest was given to a concrete physical application, namely, a system of interacting vortices, currently used as a suitable model for type-II superconductors, which exhibited such a behavior [38,43,45,46,65]. This NLFPE, that appears as a particular case of the one derived in [51,52], presents two diffusive terms: (i) A linear contribution, obtained in the usual way, i.e., by applying an additive uncorrelated thermal noise in the system [2,3,4]; (ii) A nonlinear one, characterized by a power in the probability, like in the NLFPE of [8,9], which emerged from a coarse-graining approach in the vortex-vortex interactions. From these diffusive terms, two distinct temperatures were identified, respectively, the usual thermal temperature *T*, associated with the linear contribution, and an additional temperature θ (directly related to fluctuations in the vortex positions), defined from the diffusion coefficient of the nonlinear contribution. Moreover, it was shown that θ≫T for typical type-II superconductors, so that thermal effects could be neglected, as an approximation [41]; based on this, a whole consistent thermodynamic framework was developed by considering the temperature θ and its conjugated entropy $S_{q}$, with q=2 [41,42,43,44,67,68].

Motivated by these previous investigations, in the present work we focus on a NLFPE characterized by two diffusive terms, written in the general form [46]

(3) $$\frac{\partial P(x,t)}{\partial t}=-\frac{\partial }{\partial x}\left\{A\left(x\right)\Psi \left[P\left(x,t\right)\right]\right\}+D_{1}\frac{\partial }{\partial x}\left\{\Omega_{1}\left[P\left(x,t\right)\right]\frac{\partial P(x,t)}{\partial x}\right\}+D_{2}\frac{\partial }{\partial x}\left\{\Omega_{2}\left[P\left(x,t\right)\right]\frac{\partial P(x,t)}{\partial x}\right\}\;,$$

where $D_{1}$ and $D_{2}$ represent diffusion coefficients, whereas the functionals (Ψ[P(x,t)], $\Omega_{1}\left[P\left(x,t\right)\right]$, and $\Omega_{2}\left[P\left(x,t\right)\right]$) should satisfy similar mathematical requirements as mentioned above [32,33]. One sees easily that Equation (3) may be written in the form of Equation (1), provided that

(4) $$\Omega \left[P\left(x,t\right)\right]=\frac{D_{1}}{D}\;\Omega_{1}\left[P\left(x,t\right)\right]+\frac{D_{2}}{D}\;\Omega_{2}\left[P\left(x,t\right)\right]\;.$$

Of course, the present approach can be generalized to cover situations with several (more than two) temperatures, by adding further nonlinear diffusive terms in (Equation (3)); this generalization is straightforward and will be addressed in future works.

In the analysis that follows, we discuss the physical aspects related to Equation (3), or equivalently, to Equations (1) and (4). In the next section we develop a generalized form of the H-theorem, from which the concepts of two temperatures, as well as their thermodynamically conjugated entropic forms appear, each of them associated with a given diffusion term. In Section 3 we work out the equilibrium solution of Equation (3), and we introduce a functional to be extremized, defined as a composition of the two entropic forms. We show that by choosing appropriately the Lagrange multipliers in this extremization procedure, one obtains an equation that coincides with the time-independent solution of Equation (3). In contrast to this functional, it is verified that the equilibrium solutions are not simple combinations of known equilibrium distributions, related to each diffusion contribution separately. In Section 4 we review briefly the physical application of type-II superconducting vortices, giving emphasis to its equilibrium distribution. Finally, in Section 5 we discuss the physical consequences of an equilibrium state characterized by two distinct temperatures and present our main conclusions, together with possible thermodynamic scenarios.

## 2. Generalized Forms of the H-Theorem

The H-theorem represents one of the most important results of nonequilibrium statistical mechanics, since it ensures that after a sufficiently long time, the associated system will reach an equilibrium state. In standard nonequilibrium statistical mechanics, it is usually proven by considering the BG entropy $S_{\mathrm{BG}}$, and making use of an equation that describes the time evolution of the associated probability density, like the Boltzmann, or linear FPE (in the case of continuous probabilities), or the master equation (in the case of discrete probabilities) [1,2,3,4].

Recently, the H-theorem has been extended to generalized entropic forms by using NLFPEs [7,25,26,27,28,29,30,31,32,33,34,35,36,37,38,39,40,41,42,43,44,45,46,47]. In the case of a system under a confining external potential ϕ(x) [from which one obtains the external force appearing in Equation (1), or in Equation (3), A(x)=−dϕ(x)/dx], the H-theorem corresponds to a well-defined sign for the time derivative of the free-energy functional,

(5) $$F\left[P\right]=U\left[P\right]-\gamma S\left[P\right]\;;\;U\left[P\right]=\int_{-\infty }^{\infty }\;dx\;\phi \left(x\right)P\left(x,t\right)\;,$$

where γ denotes a positive parameter with dimensions of temperature. Moreover, the entropy may be considered in the general form [32,33,35],

(6) $$S\left[P\right]=k\int_{-\infty }^{\infty }\;dx\;g\left[P\left(x,t\right)\right]\;;\;g\left(0\right)=g\left(1\right)=0\;;\;\frac{d^{2}g}{dP^{2}}\leq 0\;,$$

where *k* represents a positive constant with entropy dimensions, that can be assumed as the Boltzmann constant, whereas the functional g[P(x,t)] should be at least twice differentiable. Furthermore, the conditions that ensure normalizability of P(x,t) for all times [cf. Equation (2)] are also used in the proof of the H-theorem. For completeness, we first prove the H-theorem for the BG entropy, making use of the linear FPE.

### 2.1. The H-Theorem from the Linear Fokker-Planck Equation

It is well-established that the BG entropy, defined following Equation (6) through g[P(x,t)]=−P(x,t)ln[P(x,t)], is directly related to the linear FPE, given as a particular case of Equation (1), with the functionals Ψ[P(x,t)]=P(x,t) and Ω[P(x,t)]=1 [1,2,3,4]; moreover, in this case, one has the standard temperature in Equation (5), i.e., γ=T. Therefore, the time derivative of the free-energy functional becomes

(7) $$\begin{array}{ccc} \frac{dF}{dt} & = & \frac{\partial }{\partial t}\left(\int_{-\infty }^{\infty }dx\;\phi \left(x\right)P\left(x,t\right)+kT\int_{-\infty }^{\infty }dx\;P\left(x,t\right)\ln\left[P\left(x,t\right)\right]\right)=\int_{-\infty }^{\infty }dx\;\left[\phi (x)+kT(\ln P+1)\right]\frac{\partial P}{\partial t}\\ & = & \int_{-\infty }^{\infty }dx\;\left[\phi (x)+kT\ln P\right]\left[-\frac{\partial [A(x)P(x,t)]}{\partial x}+D\frac{\partial^{2}P\left(x,t\right)}{\partial x^{2}}\right]\;, \end{array}$$

where, in the second line we have used the particular case of Equation (1) for the time-derivative of the probability, and the normalization condition for all times, which implies on $\int_{-\infty }^{\infty }dx\left(\partial P/\partial t\right)=0$. Then, we perform an integration by parts, use the conditions of Equation (2), and assume D=kT, to obtain

(8) $$\frac{dF}{dt}=-\int_{-\infty }^{\infty }dx\;P\left(x,t\right){\left[-A\left(x\right)+\frac{kT}{P(x,t)}\frac{\partial P(x,t)}{\partial x}\right]}^{2}\leq 0\;,$$

Leading to a well-defined sign for the time-derivative of the free-energy functional. Since U[P] and S[P] are both finite for any *P*, this implies that for a finite value of γ, F[P] is bounded from below and that the reached stationary state is stable.

### 2.2. The H-Theorem from Nonlinear Fokker-Planck Equations

Considering a procedure similar to the one presented above for the general forms of the free-energy functional in Equations (5) and (6), together with the NLFPE of Equation (1), the H-theorem may be proven (see, e.g., [32,33,35]), by considering D=kγ, and imposing the condition

(9) $$-\frac{d^{2}g\left[P\right]}{dP^{2}}=\frac{\Omega [P]}{\Psi [P]}\;,$$

Which relates the entropic form to a certain time evolution. Particular entropic forms and their associated NLFPEs were explored in [32], whereas families of NLFPEs (those characterized by the same ratio Ω[P]/Ψ[P]) were studied in [35].

In what follows, we prove the H-theorem by making use of Equation (3); for that, we replace the free-energy functional of Equation (5) by

(10) $$F\left[P\right]=U\left[P\right]-\theta_{1}S_{1}\left[P\right]-\theta_{2}S_{2}\left[P\right]\;;\;U\left[P\right]=\int_{-\infty }^{\infty }\;dx\;\phi \left(x\right)P\left(x,t\right)\;,$$

where $\theta_{1}$ and $\theta_{2}$ denote positive parameters with dimensions of temperature. Similarly to Equation (6), we now define

(11) $$S_{i}\left[P\right]=k\int_{-\infty }^{\infty }\;dx\;g_{i}\left[P\left(x,t\right)\right]\;;\;g_{i}\left(0\right)=g_{i}\left(1\right)=0\;;\;\frac{d^{2}g_{i}}{dP^{2}}\leq 0\;;\;\left(i=1,2\right).$$

Hence, one obtains for the time derivative of the free-energy functional of Equation (10)

$$\begin{array}{ccc} \frac{dF}{dt} & = & \frac{\partial }{\partial t}\left(\int_{-\infty }^{\infty }dx\;\phi \left(x\right)P\left(x,t\right)-k\theta_{1}\int_{-\infty }^{\infty }dx\;g_{1}\left[P\left(x,t\right)\right]-k\theta_{2}\int_{-\infty }^{\infty }dx\;g_{2}\left[P\left(x,t\right)\right]\right)\\ & = & \int_{-\infty }^{\infty }dx\;\left[\phi \left(x\right)-k\theta_{1}\frac{dg_{1}}{dP}-k\theta_{2}\frac{dg_{2}}{dP}\right]\frac{\partial P}{\partial t}=\int_{-\infty }^{\infty }dx\;\left[\phi \left(x\right)-k\theta_{1}\frac{dg_{1}}{dP}-k\theta_{2}\frac{dg_{2}}{dP}\right]\\ & \times & \left[-\frac{\partial }{\partial x}\left\{A\left(x\right)\Psi \left[P\left(x,t\right)\right]\right\}+D_{1}\frac{\partial }{\partial x}\left\{\Omega_{1}\left[P\left(x,t\right)\right]\frac{\partial P(x,t)}{\partial x}\right\}+D_{2}\frac{\partial }{\partial x}\left\{\Omega_{2}\left[P\left(x,t\right)\right]\frac{\partial P(x,t)}{\partial x}\right\}\right]\;, \end{array}$$

where in the last line we have substituted Equation (3) for the time derivative of the probability. Hence, carrying out an integration by parts and using the conditions of Equation (2), one obtains

(12) $$\begin{array}{ccc} \frac{dF}{dt} & =- & \int_{-\infty }^{\infty }dx\;\Psi \left[P\left(x,t\right)\right]\left[-A\left(x\right)+D_{1}\;\frac{\Omega_{1}\left[P\right]}{\Psi [P]}\frac{\partial P(x,t)}{\partial x}+D_{2}\;\frac{\Omega_{2}\left[P\right]}{\Psi [P]}\frac{\partial P(x,t)}{\partial x}\right]\\ & \times & \left[-A\left(x\right)-k\theta_{1}\;\frac{d^{2}g_{1}}{dP^{2}}\frac{\partial P(x,t)}{\partial x}-k\theta_{2}\;\frac{d^{2}g_{2}}{dP^{2}}\frac{\partial P(x,t)}{\partial x}\right]\;. \end{array}$$

Remembering that the functional Ψ[P(x,t)] was defined previously as a positive quantity, for a well-defined sign of the quantity above it is sufficient to impose the conditions,

(13) $$D_{1}=k\theta_{1}\;;\;\;D_{2}=k\theta_{2}\;,$$

As well as

(14) $$-\frac{d^{2}g_{1}\left[P\right]}{dP^{2}}=\frac{\Omega_{1}\left[P\right]}{\Psi [P]}\;;\;\;-\frac{d^{2}g_{2}\left[P\right]}{dP^{2}}=\frac{\Omega_{2}\left[P\right]}{\Psi [P]}\;,$$

Extending the condition of Equation (9) for two diffusion contributions. These conditions lead to the following generalized form for the H-theorem,

(15) $$\frac{dF}{dt}=-\int_{-\infty }^{\infty }dx\;\Psi \left[P\left(x,t\right)\right]{\left[-A\left(x\right)+D_{1}\;\frac{\Omega_{1}\left[P\right]}{\Psi [P]}\frac{\partial P(x,t)}{\partial x}+D_{2}\;\frac{\Omega_{2}\left[P\right]}{\Psi [P]}\frac{\partial P(x,t)}{\partial x}\right]}^{2}\leq 0\;.$$

Like before, since F[P] [in Equation (10)] is finite for finite values of $\theta_{1}$ and $\theta_{2}$, the stationary solution satisfying (dF/dt)=0 is a stable solution.

One should notice that substituting Equation (4) in Equation (9), and using the conditions of Equation (14), one gets

(16) $$\frac{d^{2}g}{dP^{2}}=\frac{D_{1}}{D}\frac{d^{2}g_{1}\left[P\right]}{dP^{2}}+\frac{D_{2}}{D}\frac{d^{2}g_{2}\left[P\right]}{dP^{2}}\;,$$

So that integrating twice with respect to P(x,t), and using the conditions in Equations (6) and (11),

(17) $$g\left[D_{1},D_{2},D,P\left(x,t\right)\right]=\frac{D_{1}}{D}g_{1}\left[P\right]+\frac{D_{2}}{D}g_{2}\left[P\right]\;.$$

Now, integrating with respect to the variable *x*, the following functional results

(18) $$\Lambda \left[D_{1},D_{2},D,P\left(x,t\right)\right]=k\int_{-\infty }^{\infty }dx\;g\left[D_{1},D_{2},D,P\left(x,t\right)\right]=\frac{D_{1}}{D}S_{1}\left[P\right]+\frac{D_{2}}{D}S_{2}\left[P\right]\;.$$

We call attention to the fact that the functional $\Lambda [D_{1},D_{2},D,P\left(x,t\right)]$ above apparently depends on the probability, as well as on diffusion coefficients, a result that appears as a direct consequence of a NLFPE with more than one diffusion term. According to information theory, written in this form, this quantity should not be associated to an entropic form, since it violates one of its basic axioms, which states that an entropic form should depend only on the probability P(x,t) [69]. Up to the moment, this functional may be understood as a linear combination of the two entropic forms $S_{1}\left[P\right]$ and $S_{2}\left[P\right]$, with coefficients $\left(D_{1}/D\right)$ and $\left(D_{2}/D\right)$, respectively; later on, based on thermodynamic arguments, we will argue that this functional may be interpreted also as an entropic functional depending only on the probabilities.

## 3. Equilibrium Distribution

In this section we work out the stationary-state (i.e., time-independent) solution of Equation (3), as well as the equilibrium distribution that results from an extremization procedure of the functional of Equation (18). As usual, the Lagrange parameters of this later approach will be defined appropriately so that these two results coincide; based on this, in the calculations that follow we refer to an equilibrium state, described by a distribution $P_{\mathrm{eq}}\left(x\right)$.

First, let us obtain the time-independent distribution of Equation (3); for this purpose, we rewrite it in the form of a continuity equation,

(19) $$\frac{\partial P(x,t)}{\partial t}=-\frac{\partial J(x,t)}{\partial x}\;,$$

where the probability current density is given by,

(20) $$J\left(x,t\right)=A\left(x\right)\Psi \left[P\left(x,t\right)\right]-D_{1}\Omega_{1}\left[P\left(x,t\right)\right]\frac{\partial P(x,t)}{\partial x}-D_{2}\Omega_{2}\left[P\left(x,t\right)\right]\frac{\partial P(x,t)}{\partial x}\;.$$

The solution $P_{\mathrm{eq}}\left(x\right)$ is obtained by setting $J_{\mathrm{eq}}\left(x\right)=0$ (as required by conservation of probability [32]), so that

(21) $$J_{\mathrm{eq}}\left(x\right)=A\left(x\right)\Psi \left[P_{\mathrm{eq}}\left(x\right)\right]-\left\{D_{1}\Omega_{1}\left[P_{\mathrm{eq}}\left(x\right)\right]+D_{2}\Omega_{2}\left[P_{\mathrm{eq}}\left(x\right)\right]\right\}\frac{dP_{\mathrm{eq}}}{dx}=0\;,$$

Which may still be written in the form

(22) $$A\left(x\right)=\left\{D_{1}\frac{\Omega_{1}\left[P_{\mathrm{eq}}\left(x\right)\right]}{\Psi [P_{\mathrm{eq}}\left(x\right)]}+D_{2}\frac{\Omega_{2}\left[P_{\mathrm{eq}}\left(x\right)\right]}{\Psi [P_{\mathrm{eq}}\left(x\right)]}\right\}\frac{dP_{\mathrm{eq}}}{dx}\;.$$

Integrating the equation above over *x*, and remembering that the external force was defined as A(x)=−dϕ(x)/dx, one gets,

(23) $$\begin{array}{ccc} \phi_{0}-\phi \left(x\right) & = & \int_{x_{0}}^{x}dx\;\left\{D_{1}\frac{\Omega_{1}\left[P_{\mathrm{eq}}\left(x\right)\right]}{\Psi [P_{\mathrm{eq}}\left(x\right)]}+D_{2}\frac{\Omega_{2}\left[P_{\mathrm{eq}}\left(x\right)\right]}{\Psi [P_{\mathrm{eq}}\left(x\right)]}\right\}\frac{dP_{\mathrm{eq}}}{dx}\\ & = & \int_{P_{\mathrm{eq}}\left(x_{0}\right)}^{P_{\mathrm{eq}}\left(x\right)}\;\left\{D_{1}\frac{\Omega_{1}\left[P_{\mathrm{eq}}\left(x^{\prime }\right)\right]}{\Psi [P_{\mathrm{eq}}\left(x^{\prime }\right)]}+D_{2}\frac{\Omega_{2}\left[P_{\mathrm{eq}}\left(x^{\prime }\right)\right]}{\Psi [P_{\mathrm{eq}}\left(x^{\prime }\right)]}\right\}dP_{\mathrm{eq}}\left(x^{\prime }\right)\;, \end{array}$$

where $\phi_{0}\equiv \phi \left(x_{0}\right)$. Now, one uses the relations in Equation (14), and carrying the integrations,

(24) $${\left.D_{1}\frac{dg_{1}\left[P\right]}{dP}\right|}_{P=P_{\mathrm{eq}}\left(x\right)}+{\left.D_{2}\frac{dg_{2}\left[P\right]}{dP}\right|}_{P=P_{\mathrm{eq}}\left(x\right)}=\phi \left(x\right)+C_{1}\;,$$

With $C_{1}$ being a constant.

Next, we extremize the functional of Equation (18) with respect to the probability, under the constraints of probability normalization and internal-energy definition following Equation (10). For this, we introduce the functional

(25) $$I=\frac{\Lambda }{k}+\alpha \left(1-\int_{-\infty }^{\infty }dx\;P\left(x,t\right)\right)+\beta \left(U-\int_{-\infty }^{\infty }dx\;\phi \left(x\right)P\left(x,t\right)\right)\;,$$

where α and β are Lagrange multipliers. Hence, the extremization, ${\left(\delta I\right)/\left(\delta P\right)|}_{P=P_{\mathrm{eq}}\left(x\right)}=0$, leads to

(26) $${\left.\frac{D_{1}}{D}\frac{dg_{1}\left[P\right]}{dP}\right|}_{P=P_{\mathrm{eq}}\left(x\right)}+{\left.\frac{D_{2}}{D}\frac{dg_{2}\left[P\right]}{dP}\right|}_{P=P_{\mathrm{eq}}\left(x\right)}-\alpha -\beta \;\phi \left(x\right)=0\;.$$

One notices that Equations (24) and (26), resulting from the stationary-state solution of Equation (3) and the extremization of the functional of Equation (18), respectively, which in fact yield stable solutions, coincide if one chooses the Lagrange multipliers $\alpha =C_{1}/D$ and β=1/D. Moreover, one should remind that the relations in Equation (14) were used to get Equation (24).

For reasons that follow next, we impose the Lagrange multiplier $\beta =1/(D_{1}+D_{2})$, which implies on $D=D_{1}+D_{2}$; in this case, the functional of Equation (18) becomes

(27) $$\Lambda \left[\theta_{1},\theta_{2},P\left(x,t\right)\right]=\frac{\theta_{1}}{\theta_{1}+\theta_{2}}S_{1}\left[P\right]+\frac{\theta_{2}}{\theta_{1}+\theta_{2}}S_{2}\left[P\right]\;,$$

where we have used the temperature definitions of Equation (13). This particular choice for the Lagrange multiplier β yields a functional $\Lambda [\theta_{1},\theta_{2},P\left(x,t\right)]$, given by a linear combination of the two entropic forms $S_{1}\left[P\right]$ and $S_{2}\left[P\right]$ with well-defined coefficients, representing one of the main novelties of this investigation; it presents important properties, listed below.

(i) The corresponding coefficients are both in the interval [0,1] and their sum gives unit, so that they may be interpreted as probabilities related to the contribution of each entropic form to the functional $\Lambda [\theta_{1},\theta_{2},P\left(x,t\right)]$; in this way, the quantity in Equation (27) can be understood as a mean value.

(ii) When one temperature prevails with respect to the other one, e.g., $\theta_{1}\gg \theta_{2}$, the resulting functional becomes essentially the entropic form associated to this temperature, i.e., $\Lambda \approx S_{1}\left[P\right]$, like considered in the physical application explored in [38,43,45,46,65].

(iii) In Equation (17), one has

(28) $$g\left[\theta_{1},\theta_{2},P\right]=\frac{\theta_{1}}{\theta_{1}+\theta_{2}}g_{1}\left[P\right]+\frac{\theta_{2}}{\theta_{1}+\theta_{2}}g_{2}\left[P\right]\;,$$

So that the conditions of Equation (11) yield $g\left[\theta_{1},\theta_{2},0\right]=g\left[\theta_{1},\theta_{2},1\right]=0$, for arbitrary values of $\theta_{1}$ and $\theta_{2}$.

(iv) The concavity of the functional $\Lambda [\theta_{1},\theta_{2},P\left(x,t\right)]$ with respect to the probability is well defined; indeed, Equation (16) leads to

(29) $$\frac{d^{2}g\left[\theta_{1},\theta_{2},P\right]}{dP^{2}}=\frac{\theta_{1}}{\theta_{1}+\theta_{2}}\frac{d^{2}g_{1}\left[P\right]}{dP^{2}}+\frac{\theta_{2}}{\theta_{1}+\theta_{2}}\frac{d^{2}g_{2}\left[P\right]}{dP^{2}}\leq 0\;,$$

where the inequality comes as a direct consequence of the conditions in Equation (11). In addition to this, due to the properties of the coefficients (described in (i) above), the second derivative on the left-hand-side should be in between the two second derivatives on the right-hand side.

## 4. Physical Application: Type-II Superconducting Vortices

One case of interest in Equation (3) corresponds to the competition of two power-like diffusive terms, given by the NLFPE [46]

(30) $$\frac{\partial P(x,t)}{\partial t}=-\frac{\partial }{\partial x}\left\{A\left(x\right)P\left(x,t\right)\right]\}+D_{1}\;\frac{\partial^{2}P^{\mu_{1}}\left(x,t\right)}{\partial x^{2}}+D_{2}\;\frac{\partial^{2}P^{\mu_{2}}\left(x,t\right)}{\partial x^{2}}\;,$$

where $\left(D_{1},\mu_{1}\right)$ and $\left(D_{2},\mu_{2}\right)$ correspond to coefficients and exponents related to each diffusion contribution; comparing with Equation (3), one has that

(31) $$\begin{array}{ccc} \Psi [P(x,t)] & = & P(x,t)\;,\\ \Omega_{1}\left[P\left(x,t\right)\right] & = & \mu_{1}{\left[P\left(x,t\right)\right]}^{\mu_{1}-1}\;;\;\Omega_{2}\left[P\left(x,t\right)\right]=\mu_{2}{\left[P\left(x,t\right)\right]}^{\mu_{2}-1}\;. \end{array}$$

Therefore, the integrations in Equation (14) lead to

(32) $$S_{1}\left[P\left(x,t\right)\right]=k\int_{-\infty }^{\infty }\;dx\;\frac{P\left(x,t\right)-{\left[P\left(x,t\right)\right]}^{\mu_{1}}}{\mu_{1}-1}\;;\;S_{2}\left[P\left(x,t\right)\right]=k\int_{-\infty }^{\infty }\;dx\;\frac{P\left(x,t\right)-{\left[P\left(x,t\right)\right]}^{\mu_{2}}}{\mu_{2}-1}\;,$$

Which compose the functional in Equation (27). By comparing this functional with previous ones [e.g., cf. Equation (23) of [46]], the main novelty herein concerns the coefficients of each entropic form, written in the form of probabilities. In this way, the equilibrium equation [cf. Equation (24)] becomes

(33) $$\frac{D_{1}\mu_{1}}{\mu_{1}-1}\;P_{\mathrm{eq}}^{\mu_{1}-1}\left(x\right)+\frac{D_{2}\mu_{2}}{\mu_{2}-1}\;P_{\mathrm{eq}}^{\mu_{2}-1}\left(x\right)=-\phi \left(x\right)+C_{1}\;.$$

Recently, a special interest was given to a physical application, namely, a system of interacting vortices, used as a suitable model for type-II superconductors [38,43,45,46,65]. For this particular system, in the second diffusive contribution one has $\mu_{2}=2$ and $D_{2}=k\theta$, where an effective temperature θ was related to the density of vortices [41]. The first diffusive term comes from a standard uncorrelated thermal noise, leading to the linear contribution ($\mu_{1}=1$) and $D_{1}=kT$. The functional of Equation (27) becomes

(34) $$\begin{array}{ccc} \Lambda [\theta ,T,P(x,t)] & = & \frac{T}{T+\theta }\;S_{1}\left[P\left(x,t\right)\right]+\frac{\theta }{T+\theta }\;S_{2}\left[P\left(x,t\right)\right]\\ & = & -\frac{kT}{T+\theta }\int_{-\infty }^{\infty }\;dx\;P\left(x,t\right)\ln P\left(x,t\right)+\frac{k\theta }{T+\theta }\int_{-\infty }^{\infty }\;dx\;\left\{P\left(x,t\right)-{\left[P\left(x,t\right)\right]}^{2}\right\}\;, \end{array}$$

Which differs from previous ones [e.g., Equation (17) of [38], or Equation (47) of [46]] in the choices for the coefficients. Particularly, with respect to the result of [38], one has now a concrete proposal for the quantity in the denominator of these coefficients, which appeared herein from an appropriate choice of the Lagrange multiplier β.

In the present case, Equation (33) becomes

(35) $$D_{1}\ln P_{\mathrm{eq}}\left(x\right)+2D_{2}P_{\mathrm{eq}}\left(x\right)=C^{\prime \prime }-\phi \left(x\right)\;,$$

Which can be written as

(36) $$\frac{2D_{2}\;P_{\mathrm{eq}}\left(x\right)}{D_{1}}\exp\left(\frac{2D_{2}}{D_{1}}P_{\mathrm{eq}}\left(x\right)\right)=\frac{2D_{2}}{D_{1}}\exp\left(\frac{C^{\prime \prime }-\phi \left(x\right)}{D_{1}}\right)\;.$$

where $C^{\prime \prime }=C_{1}-D_{1}+D_{2}$.

In the equation above one identifies the form $Xe^{X}=Y$, which defines the implicit *W*-Lambert function, such that X=W(Y) (see, e.g., [70]). Therefore,

(37) $$P_{\mathrm{eq}}\left(x\right)=\frac{D_{1}}{2D_{2}}W\left\{\frac{2D_{2}}{D_{1}}\exp\left(\frac{C^{\prime \prime }-\phi \left(x\right)}{D_{1}}\right)\right\}\;.$$

Choosing a harmonic confining potential, the distribution above interpolates between two well-known limits, namely, the Gaussian distribution $\left(D_{1}\gg D_{2}\right)$ and the parabola, i.e., *q*-Gaussian distribution with q=2$\left(D_{1}\ll D_{2}\right)$; moreover, both parameters $D_{1}$ and $D_{2}$ affect directly the width of the distribution, consistently with the temperature definitions of Equation (13), in the sense that larger values of these parameters produce larger widths [46].

Hence, considering $\phi \left(x\right)=\alpha x^{2}/2$(α>0), and the temperature definitions of Equation (13) for the present case, i.e., $D_{1}=kT$ and $D_{2}=k\theta$, g one has

(38) $$P_{\mathrm{eq}}\left(x\right)=\frac{T}{2\theta }W\left\{\frac{2\theta }{T}\exp\left(\frac{C^{\prime \prime }}{kT}-\frac{\alpha x^{2}}{2kT}\right)\right\}\;,$$

**Figure 1:**
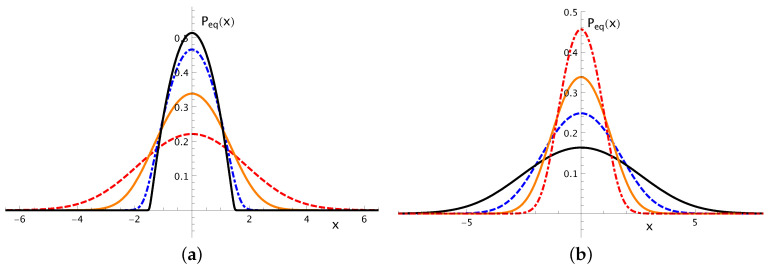
The equilibrium-state distribution $P_{\mathrm{eq}}\left(x\right)$ of Equation (38) is represented versus *x* by considering k=1 and special choices of its parameters. (**a**) Fixing α=θ=1 and increasing values of *T* [T=0.02,0.2,1.0, and 3.0 (from top to bottom)], showing the crossover from the parabolic to the Gaussian behavior; (**b**) Fixing T=θ=1 and increasing values of α [α=0.2,0.5,1.0, and 3.0 (from bottom to top)]. The parameter $C^{\prime \prime }$ is found in each case by imposing normalization for $P_{\mathrm{eq}}\left(x\right)$.

Which is illustrated in Figure 1 for typical choices of its parameters. The crossover between the parabolic behavior (T≪θ) to the Gaussian distribution (T≫θ) is shown in Figure 1a, where we present equilibrium distributions for α=θ=1 and increasing values of *T* (from top to bottom). The former limit is important for an appropriate description of a type-II superconducting phase, where one finds strongly-interacting vortices [41,43], whereas in the latter limit one approaches the behavior of a system of weakly-interacting particles [45]. In Figure 1b we present equilibrium distributions by considering T=θ=1 (i.e., in between the two limits mentioned above) and increasing values of α. As expected, the parameter α, which represents the strength of the confining potential, is directly related to the confining of the vortices, affecting the distribution width, in the sense that larger values of α correspond to smaller distribution widths. In the first limiting behavior, a whole consistent thermodynamic framework was developed by neglecting thermal effects, considering the temperature θ, its conjugated entropy $S_{q}$ (with q=2), the parameter α, and introducing its conjugated parameter σ [41,42,43,44,67,68]. At this point, it is important to emphasize that the two temperatures exist in an equilibrium state (there is no flux), presenting different physical meanings. The usual temperature *T* is related to the thermal noise, and may be changed through heat transfers to (or from) the system, whereas the temperature θ is related to the density of vortices, and can be varied by monitoring an applied magnetic field. Even with these two temperatures, with different physical meanings, there is no nonequilibrium behavior, so that the thermodynamic formalism can be considered.

Recently, by analyzing short-range power-law interactions and introducing correlations among particles, a coarse-graining approach has led to a more general NLFPE, extending the previous results to a wider range of values of *q* [71]. Motivated by this, a consistent thermodynamic framework was proposed for *q*-Gaussian distributions characterized by a cutoff, under similar conditions [47].

Next, we discuss further the most general physical situation where both temperatures may be of the same order of magnitude. Following previous analyses [42,43,44,67,68], we consider that the parameter α may be varied continuously, and that these variations are related to a work contribution in a first-law proposal.

## 5. Discussion and Conclusions

In this section we analyze possible thermodynamical scenarios describing the two-temperature equilibrium state (temperatures $\theta_{1}$ and $\theta_{2}$) discussed in Section 3. Considering the free-energy functional of Equation (10), one may define two different types of heat-like contributions, related to variations in each entropic contribution, namely, $\delta Q_{1}=\theta_{1}dS_{1}$, and $\delta Q_{2}=\theta_{2}dS_{2}$. Moreover, inspired by the physical example of Section 4, one can consider the parameter α as some controllable external field associated with work, which affects directly the volume occupied by the particles; in this way, following previous investigations, we introduce an infinitesimal work contribution, δW=σdα [42,43,44,67,68]. Hence, infinitesimal variations in the internal energy *U* can be associated to these proposals for infinitesimal work and heat contributions, yielding an equivalent to the first law,

(39) $$dU=\delta Q_{1}+\delta Q_{2}+\delta W=\theta_{1}dS_{1}+\theta_{2}dS_{2}+\sigma d\alpha \;,$$

where the two temperatures may be obtained from

(40) $$\theta_{1}={\left(\frac{\partial U}{\partial S_{1}}\right)}_{S_{2},\;\alpha }\;;\;\theta_{2}={\left(\frac{\partial U}{\partial S_{2}}\right)}_{S_{1},\;\alpha }\;.$$

One should remind that, since the present results follow from a NLFPE, the thermodynamic quantities ($U,S_{1},S_{2},\alpha$), presenting infinitesimal changes in Equation (39), refer to one particle of the system. Furthermore, σ corresponds to the parameter thermodynamically conjugated to α, to be determined from an equation of state; considering Equation (39), one has two equivalent ways to calculate σ,

(41) $$\sigma =-\theta_{1}{\left(\frac{\partial S_{1}}{\partial \alpha }\right)}_{U,\;S_{2}}\;;\;\sigma =-\theta_{2}{\left(\frac{\partial S_{2}}{\partial \alpha }\right)}_{U,\;S_{1}}\;,$$

By keeping fixed one of the two entropic forms in each case. Summing these two equations, one has

(42) $$\sigma =-\frac{1}{2}\left[\theta_{1}{\left(\frac{\partial S_{1}}{\partial \alpha }\right)}_{U,\;S_{2}}+\theta_{2}{\left(\frac{\partial S_{2}}{\partial \alpha }\right)}_{U,\;S_{1}}\right]\;,$$

Which relates the quantities ($\theta_{1},\theta_{2},\sigma ,\alpha$), representing the equation of state of the system.

Now, we assume that a given thermodynamical transformation may occur in such a way that variations in the two entropic forms lead to a variation in the functional Λ of Equation (27) following

(43) $$\left(\theta_{1}+\theta_{2}\right)d\Lambda =\theta_{1}dS_{1}+\theta_{2}dS_{2}=\delta Q_{1}+\delta Q_{2}\;.$$

In fact, the result above holds in general (not only for a specific transformation) if one considers Λ as a thermodynamic quantity whose natural variables are $S_{1}$ and $S_{2}$, i.e., $\Lambda \equiv \Lambda (S_{1},S_{2})$. This is justified by the argument that, in a many-particle system, both entropic forms $S_{1}$ and $S_{2}$ should be extensive quantities, and so, extensivity of $\Lambda (S_{1},S_{2})$ follows by imposing Λ to be a homogeneous function of first degree of $S_{1}$ and $S_{2}$,

(44) $$\Lambda \left(\lambda S_{1},\lambda S_{2}\right)=\lambda \Lambda \left(S_{1},S_{2}\right)\;,$$

where λ is a positive real number. In this way, one may write the first-law in Equation (39) in the form,

(45) $$dU=(\theta_{1}+\theta_{2})d\Lambda +\sigma d\alpha \;,$$

From which one identifies $\left(\theta_{1}+\theta_{2}\right)$ as the parameter thermodynamically conjugated to Λ. Therefore, another temperature (to be called γ) may be defined, leading to the fundamental equation of thermodynamics,

(46) $$\gamma =\theta_{1}+\theta_{2}={\left(\frac{\partial U}{\partial \Lambda }\right)}_{\alpha }\;,$$

Whereas the equation of state may be expressed as

(47) $$\sigma =-\gamma {\left(\frac{\partial \Lambda }{\partial \alpha }\right)}_{U}\;.$$

One should notice that the temperature parameter γ above now appears as a concrete proposal of this quantity in the two-entropic functional of previous works [e.g., Equation (17) of [38]].

Consequently, from the statistical point of view, the dependence of Λ on the entropic forms $S_{1}\left[P\right]$ and $S_{2}\left[P\right]$ imply on Λ≡Λ[P]; furthermore, the effective-temperature definition of Equation (46) and the first law in the form of Equation (45) are consistent with a free-energy functional similar to Equation (5),

(48) $$F\left[P\right]=U\left[P\right]-\gamma \Lambda \left[P\right]\;;\;U\left[P\right]=\int_{-\infty }^{\infty }\;dx\;\phi \left(x\right)P\left(x,t\right)\;,$$

So that results for systems with a single temperature and entropic form apply to the pair of conjugated variables (γ,Λ) defined above. Particularly, the H-theorem for the NLFPE with two diffusive terms in Equation (3) may be proven by considering the functional of Equation (4), leading to the relation in Equation (9), as well as the equivalence of the stationary-state solution of the NLFPE and the extremization of the functional Λ[P] (see, e.g., [46]). This means that the stationary-state solution of the NLFPE yields the same solution as the MaxEnt principle, allowing us to assert that this is an equilibrium solution.

To summarize, we have studied a general physical situation of a system characterized by two distinct temperatures, $\theta_{1}$ and $\theta_{2}$, which may be of the same order of magnitude, and their thermodynamically conjugated entropic forms, $S_{1}$ and $S_{2}$, respectively. This study was motivated by recent investigations concerning type-II superconducting vortices, where two entropic forms corresponding to $S_{q}$ with q=2 and BG entropy $S_{\mathrm{BG}}$, conjugated to temperatures θ and *T*, appeared. However, in this case, it was shown that an approximated physical description could be developed by neglecting thermal effects, based on the fact that along a typical type-II superconducting phase, the two associated temperatures are significantly different in magnitude, i.e., θ≫T. In the present work we have assumed that the temperatures should present physically distinct properties, like in the case of type-II superconductors, where they are associated respectively, to fluctuations in velocities and positions of the particles; in this way, an equilibrium state may be attained, with a temperature $\theta_{1}$ having a different physical meaning than the temperature $\theta_{2}$. The procedure was based on a nonlinear Fokker-Planck equation, where the temperatures appeared as coefficients of different diffusion contributions. We have proven an H-theorem, relating such a Fokker-Planck equation to a sum of two entropic forms, each of them associated with a given diffusion term. Due to the H-theorem, the corresponding stationary state is considered as an equilibrium state, characterized by two temperature parameters, $\theta_{1}$ and $\theta_{2}$, and their associated entropic forms, $S_{1}$ and $S_{2}$. Particularly, a free-energy functional, together with a first-law proposal, define a four-dimensional space ($\theta_{1},\theta_{2},S_{1},S_{2}$) where physical transformations may take place, in such a way to develop a consistent thermodynamical framework. We have also introduced a functional $\Lambda \left[P\right]\equiv \Lambda (S_{1}\left[P\right],S_{2}\left[P\right])$, together with a thermodynamically conjugated temperature parameter $\gamma (\theta_{1},\theta_{2})$, so that an alternative physical description is proposed in terms of these pair of variables. We have shown that the functional Λ[P] presents properties characteristic of an entropic form, e.g., it depends only on the probability distribution, and it presents the appropriated concavity sign.

The above-mentioned proposals, and particularly the thermodynamic properties of a system with two distinct temperatures, together with their conjugated entropies, represent open problems of relevant interest that require further investigations from both theoretical and experimental points of view. Among potential candidates in nature, one could mention: (i) Systems of particles interacting repulsively, for which a coarse-graining procedure on the interactions lead to a diffusion contribution in a Fokker-Planck equation of the same order of magnitude as the standard linear diffusion term, associated with an additive uncorrelated thermal noise; (ii) Anomalous-diffusion phenomena in porous media constituted by more than one type of material, or even random porous media.
